# Economic assessment of using Bermudagrass stockpiling and annual cereal pasture to extend grazing in cow-calf operations

**DOI:** 10.1093/tas/txae067

**Published:** 2024-04-22

**Authors:** Jacob Sestak, Jon T Biermacher, B Wade Brorsen, James K Rogers

**Affiliations:** BancFirst, McCloud, OK 74851, USA; Department of Agribusiness and Applied Economics, North Dakota State University, Fargo, ND 58108-6050, USA; Department of Agricultural Economics, Oklahoma State University, Stillwater, OK 74078-6026, USA; North Central Research Extension Center, North Dakota State University, Minot, ND 58701, USA

**Keywords:** bermudagrass grazing, economics, rotational stocking, season extension, wheat forage

## Abstract

Bermudagrass (*Cynodon dactylon* L.) stockpiling and cool-season annual pastures can extend grazing seasons in cow-calf operations and reduce winter feeding costs, but less is known about how these practices interact and their effect on producer profitability. Data from a completely randomized-design experiment in South-Central Oklahoma were collected on three grazing systems for cows and calves: bermudagrass pasture (CONTROL), stockpiled bermudagrass and interseeded cool-season pasture (SPINT), and stockpiled bermudagrass plus cropland no-till seeded with a summer cover-crop followed by cool-season annuals (SPCROP). A mixed model was used to estimate the effects of grazing system on weaning weights, total hay, and total range cubes (crude protein [CP] = 30%) fed in each system. Enterprise budgeting was used to calculate the expected net return of each system. Weaning weight did not vary between systems (*P *= 0.6940), resulting in similar revenues. Relative to other treatments, the quantity of cubes fed in the CONTROL system were significantly higher (*P* < 0.0001) while hay fed was significantly higher in the SPCROP system (*P *= 0.0036). Increased machinery costs, seed costs, and fertilization requirements in bermudagrass stockpiling, interseeding, and cropland production outweighed the cost savings associated with less feeding. Total costs were $446 ha^−1^ ($722 hd^−1^), $451 ha^−1^ ($732 hd^−1^), and $553 ha^−1^ ($895 ha^−1^) for the CONTROL, SPINT, and SPCROP systems, respectively. Overall, the CONTROL system was $3.13 ha^−1^ ($5.08 hd^−1^) and $98.91 ha^−1^ ($160.10 hd^−1^) more profitable than the SPINT and SPCROP systems.

## Teaser

Efforts to extend the cow-calf grazing season with bermudagrass pasture succeeded in reducing feeding of protein cubes, yet reduced profits.

## Introduction

In cow-calf operations of the southern great plains, cattle commonly graze bermudagrass (*Cynodon dactylon* L.) pastures and diets are supplemented with hay and protein cubes in the winter. Winter feeding costs account for the majority of expenses in cow-calf operations (Lomas et al., 2000; Karn et al., 2005; Lardy and Caton, 2010; Hibbard et al., 2021). Warm-season grass stockpiling, grazing annual summer and winter crops, and interseeding cool-season annual grasses into perennial pastures have been promoted as ways to extend grazing seasons and reduce feeding costs (Utley and McCormick, 1978; Gunter et al., 2003; Troxel et al., 2014). As suggested by Benson (2010), grazing season extension can reduce the need for stored and purchased feedstuffs and decrease forage harvesting and feeding machinery and labor costs. This study tests the hypotheses that grazing season extension through bermudagrass stockpiling and warm and cool-season forages can reduce feed costs and increase net returns in cow-calf operations of the southern great plains.

As a cool-season annual, winter wheat (*Triticum aestivium* L.) can reduce winter feeding costs by extending the grazing season (Dillard et al., 2018; Mullenix & Rouquette, 2018). Cool-season grasses alone have increased the grazing season by up to 90 total days (Hoveland et al., 1978; Rouquette 2017; Mullenix & Rouquette, 2018). Wheat planted in mid-September typically increases forage mass until late November, with a slight decrease until late January, followed by the greatest herbage accumulation through mid-May before senescence (Rouquette, 2015). Grazing winter wheat can reduce the need for both supplementary forage and protein cubes during the winter and spring. In a review of cool- and warm-season grazing systems by Dillard et al. (2018), cool-season annual forage crops were found to provide quality forage biomass when perennial forage species were lacking and reduced the need for stored feed during winter months. A multi-year grazing experiment at Oklahoma State University in the early 1990s concluded that fall-calving cows grazing wheat pasture on alternate days and calves with continuous calf creep access to wheat pasture increased calf gains and decreased the need for additional supplementation to the cows (Apple et al., 1991, 1993).

Warm-season perennial grasses decrease herbage accumulation and nutritive value after the first frost, which occurs typically in mid-November. Stockpiling forage is the act of deferring grazing and allowing warm-season forage to reach maximum forage mass. Stockpiling forage has been shown to reduce winter feed costs (Hitz & Russell, 1998; Riesterer et al., 2000; Scarbrough et al., 2001; Johnson et al., 2002; Wheeler et al., 2002; Beck et al., 2016). In a multi-year 300-d grazing experiment, Troxel et al. (2014) found total grazing days were extended to 316 ± 20.4 d by using rotational stocking systems with stockpiled bermudagrass. Additionally, Senturklu and Landblom (2017) concluded that a stockpiled grass and crop residue grazing system reduced cow wintering costs relative to conventional grazing systems. In another example supporting stockpiling, Poore and Drewnoski (2010) found that stockpiled forages met minimum nutrient requirements for early gestation or non-lactating cows in tall fescue (*Festuca arundinacea* Schreb.) paddocks. Stockpiled forages, especially bermudagrass stockpiles, have less nutritive value than non-dormant warm-season grasses. Respiration, leaf drop, and the leaching of nutrients cause declines in the nutritive value of stockpiled forages (Ocumpaugh & Matches, 1977; Matches & Burns, 1995; Baron et al., 2004).

Due to additional challenges associated with cow-calf research relative to stocker research, most overwinter forage grazing studies have focused on stocker operations (Rouquette, 2015). While grazing season extension practices have been economical in stocker cattle, cow-calf operations have not had the same level of investigation. The objectives of this study were to (1) estimate the effects of grazing system on calf birth weight (BWT), calf weaning weight, cow body condition score (BCS) prior to breeding, total kg of hay fed per month, and total kg of cube supplement fed per month, (2) discover the grazing system with the greatest economic return to land, management, and farm overhead, and (3) determine how sensitive base-case results are by *ceteris paribus* changes in fertilizer price, feed price (hay and cubes), labor price, and time devoted to feeding.

## Materials and methods

### Experimental Description

All animal procedures in the following study were performed under the recommendations of the Guide for Care and Use of Agricultural Animals and Research and Teaching and were approved by the Noble Research Institute’s Animal Care and Use Committee (IACUC) prior to the initiation of the study in 2016.

Data were from a 4-yr completely randomized-design grazing experiment (2016 to 2020) conducted in south central Oklahoma at the Noble Research Institute’s Pasture Research and Demonstration Farm near the community of Ardmore, Oklahoma, USA (34°13ʹ00.9″N, 97°12ʹ31.1″W) on nine 16 ha paddocks. Each paddock had three sub-paddocks to allow rotational stocking. Soils within the study area consist of Chickasha loam (fine-loamy, mixed, active, and thermic Udic Argiustolls) and Renfrow silt loam (fine, mixed, superactive, and thermic Udertic Paleustolls). Assigned systems include: (1) 16 ha of Midland bermudagrass pasture supplemented with bermudagrass hay and 30% range cubes (Table 1); CONTROL), (2) 16 ha of bermudagrass pasture with 4 ha used for bermudagrass stockpile (25% of paddock) and 4 ha interseeded with wheat (*Triticum aestivum* cv. Gallagher (2016 to 2017), NF101 (2018 to 2019), Oklahoma Genetics, Inc.; 25% of paddock; SPINT), and (3) 13 ha of bermudagrass pasture with 4 ha of the bermudagrass stockpiled (25% of paddock) plus 3 ha of cropland consisting of a summer cover crop (CC) followed by wheat (SPCROP). Each grazing system was replicated three times each year over the course of the study. Cow performance variables recorded included body weight (BW) and BCS (Richards, et al., 1986) prior to breeding, BW and BCS at calving and weaning, and BW and BCS post-breeding. Calf performance variables of calf birth weight (BWT), weight prior to breeding of dam, and calf weaning weight were recorded.

**Table 1.** Nutrient composition of range cubes used as a supplement during the 4-yr study

Content	Analysis
Crude protein, not less than	30.00%
Total digestible nutrients (TDN)	70.00%
Crude fat, not less than	2.50%
Crude fiber, not more than	10.00%
Calcium, not less than	0.50%
Calcium, not more than	1.00%
Phosphorus, not less than	0.75%
Salt, not less than	0.75%
Salt, not more than	1.25%
Potassium, not less than	1.30%
Vitamin A, not less than	10,000 IU/LB

### Agronomic Practices

The chronology of forage and cattle management practices is reported in Table 2. After calving began, a soil sample from each paddock was obtained to determine fertility requirements by randomly taking 12 to 15 soil cores to a 15 cm depth, and then combining the cores for a soil sample that represented the paddock area.

**Table 2.** Chronology of pasture and cattle management activities by month and grazing system

	Grazing system^*^			
Production activity	Month	CONTROL	SPCROP	SPINT
Cowherd pre-breeding vaccinations, de-worm and external parasite control	May	x	x	x
Annual weed control on bermudagrass	May	x	x	x
Chemical burndown prior to CC planting No-till drill cover crop	May May		x x	
Apply N, P, K, and lime to bermudagrass	May	x	x	x
Breeding period	May to July	x	x	x
Apply N to bermudagrass for stockpile	August		x	x
Chemically burn down cover crop	September		x	
No-till drill wheat seed	September		x	x
De-worm and apply external parasite control to cowherd	October	x	x	x
De-worm, vaccinate, and apply external parasite control to calves	October	x	x	x
Apply N, P, K to wheat	October		x	x
Feed hay	December to April	x	x	x
Feed protein cubes	December to March	x	x	x
Feed protein cubes	April	x	x	

^*^CONTROL is bermudagrass pasture and conventional hay and cube feeding; SPINT bermudagrass pasture with bermudagrass stockpile and interseeded wheat; SPCROP is bermudagrass with bermudagrass stockpile and cropland summer and winter pasture.

Fertilizer applications according to soil test recommendations began in May, with each paddock receiving equal applications of nitrogen (N) in the form of urea at a rate of 112 kg ha^−1^. Phosphorus (P_2_O_5_), potassium (K_2_O), and lime (100% ECCE equivalent) applications were completed based on soil test results obtained from paddock soil samples (Table 3). Urea (46-0-0), diammonium phosphate (18-46-0), and potash (0-0-60) were sources for N, P_2_O_5_, and K_2_O, respectively. Stockpiled bermudagrass and wheat received an additional top dress of 56 kg ha^−1^ N and 67 kg ha^−1^ N each year, respectively, in both the SPINT and SPCROP systems.

**Table 3.** Levels of phosphorus (P_2_O_5_), potassium (K_2_O), and lime (100% ECCE) applied by year and replication

System	Year	Replication	P_2_O_5_ (kg ha^−1^)	K_2_O (kg ha^−1^)	Lime (MT ha^−1^)
Control	2016	1,2,3	52		
Control	2017	1,3		34	
Control	2017	1,2,3	52		4.48
SPINT	2016	1,2,3	52	56	
SPINT	2017	1,2,3	52	34	4.48
SPCROP	2016	2	52		
SPCROP	2016	1,3		56	
SPCROP	2017	2,3	52		
SPCROP	2017	1,2,3		34	
SPCROP	2017	1,3			4.48

All pastures were sprayed with picloram-based herbicides (Grazon P + D, Corteva Agroscience, Wilmington, Delaware, USA) at a rate of 3.5 L ha^−1^ in 2016 and 2.8 L ha^−1^ in 2017, 2018, and 2019 to control broadleaf annual weeds. In the cropland portion of the SPCROP system, glyphosate (Ranger Pro, Monsanto, Creve Coeur, MO USA) was applied in September prior to seeding annual winter pasture and again in May prior to planting summer CCs. A rate of 2.1 L ha^−1^ was applied in 2016 and 2017, and a glyphosate plus 2,4-d dicamba (Brash, WinField United, Arden Hills, Minnesota, USA) mix was applied at 1.4 L ha^−1^ 2,4-d dicamba and 2.1 L ha^−1^ glyphosate (40% dicamba and 60% glyphosate) in 2018 and 2019.

Bermudagrass stockpile hectares in the CONTROL, SPINT, and SPCROP systems were over-seeded with annual ryegrass (*Lolium multiflorum* L. cv. Marshall) at a bulk seeding rate of 22.4 kg ha^−1^ in mid-August of year one. Note that annual ryegrass was introduced into these paddocks many years prior to the start of this study. To ensure that each paddock had similar forages in each paddock, annual ryegrass seed was added. Because annual ryegrass reaches its peak production in this environment in early spring, it does not compete with wheat in the fall. Following year one, annual ryegrass did not require further seeding as it successfully self-propagated. In the SPCROP and SPINT systems, wheat was no-till drilled in mid-September of each year. Prior to planting, the SPINT paddocks were grazed to a 15 cm forage height and the 3 ha of cropland in the SPCROP system was sprayed either with glyphosate or a glyphosate plus 2,4-d dicamba mix to terminate the residual CC. Wheat was no-till planted (John Deere 1,590 no-till drill + John Deere 6150R tractor, John Deere, Moline, IL) to an approximate seeding depth of 2.5 cm. ‘Gallagher’ wheat was planted in 2016 and 2017 and ‘NF101’ wheat was planted in 2018 and 2019. Both varieties were planted at a rate of 128 kg ha^−1^.

A summer CC mixture of 40% legume and 60% warm-season grass was planted for the SPCROP system in late May at a rate of 33.63 kg ha^−1^. In 2016 and 2017 the CC mixture included 6.73 kg ha^−1^ iron and clay cowpeas, 6.73 kg ha^−1^ soybeans (*Glycine Max* L.), 3.36 kg ha^−1^ sunn hemp (*Crotalaria juncea* L.), 3.36 kg ha^−1^ pearl millet (*Pennisetum glaucum* L.), 2.24 kg ha^−1^ German (foxtail) millet (*Panicum italicum* L.), 2.24 kg ha^−1^ browntop millet (*Urochloa ramosa* L.), 4.48 kg ha^−1^ brown midrib grazing corn (*Zea mays* L.), and 3.36 kg ha^−1^ buckwheat (*Fagopyrum esculentum* L.). The mix was based on unpublished research at the Noble Research Institute which had shown that each had done well when grown individually. Soybeans, German millet, browntop millet, grazing corn, sunn hemp, and buckwheat were unable to compete and did not persist in the mixture and were removed. Pearl millet and cowpeas, which did persist, were maintained in all years of the study. Okra (*Abelmoschus esculentus* L. cv. Clemson Spineless) was added in 2018 and 2019 as a broadleaf component. The CC mix in 2018 and 2019 was 6.73 kg ha^−1^ pearl millet, 3.36 kg ha^−1^ okra, and 23.54 kg ha^−1^ cowpeas.

Rainfall amounts were consistently above 30-yr average levels for the experimental area in the late winter/early spring months, below average in summer months, and variable in the fall/early winter months. Recorded rainfall for the experimental area is given in Table 4. Dry spans, especially those associated with summer CCs, hindered crop growth and performance.

**Table 4.** Rainfall in centimeters by month and year at the Pasture Research and Demonstration Farm located near Ardmore, Oklahoma

Month/Year	2016	2017	2018	2019	2020	Avg.	30-yr avg.
January	1.65	8.41	0.38	5.87	8.86	5.03	4.19
February	4.14	6.5	18.19	6.5	9.47	8.96	5.31
March	8.92	2.79	8.76	6.12	13.69	8.06	6.71
April	20.98	7.7	5.1 1	15.11	6.45	12.56	8.38
May	13.44	13.72	15.34	11.48	—	13.50	14.1
June	9.47	5.92	6.81	13.72	—	8.98	10.69
July	2.24	13.51	5.16	1.47	—	5.60	7.16
August	5.03	17.5	13.89	15.72	—	13.04	6.99
September	6.2	5.23	23.14	10.41	—	11.25	8.08
October	4.04	2.59	32.26	13.61	—	13.13	10.9
November	5.38	0.15	1.35	7.54	—	3.61	5.69
December	2.08	4.6	13.13	2.08	—	5.47	5.79
Total	83.57	88.62	138.41	109.63	38.47	109.16	93.99
Source: www.mesonet.org							

### Cow, Breeding Bull, and Calf Management

In March of 2015, prior to the initiation of the study, 90 high percentage (75%) Angus (*Bos taurus*), mature (4.4 ± 0.79 yr of age) cows were selected from the Noble Research Institute’s cowherd and assembled at the Pasture Research and Demonstration Farm (previously described) for breeding. In May 2015, all cows went through an estrous synchronization program (7-d CIDR-PG; controlled internal drug release, Pfizer, New York, NY; Lutulyse, Zoetis, Parsippany-Troy Hills, NJ) followed by a timed artificial insemination to a Hereford bull. All 90 cows were then exposed to four Hereford bulls for 60 d. Bulls were 18 mo of age in year one and were maintained throughout the study. One bull, due to a foot injury was replaced with a bull of similar age and breeding. Two additional bulls were added in 2016. After 2015, breeding was natural service only with cows being exposed from mid-May to mid-July and calving mid-February to the end of April. The same bulls were assigned to the same group of cows over the course of the study. Over the breeding period and prior to the start of the study in October 2015, cows were rotationally stocked across all treatment paddocks. In October 2015, cows with an initial BW of 544 ± 59.9 kg and BCS (1 to 9-point scale with 1 = extremely thin and 9 = obese) of 5.5 ± 0.6 were stratified by weight and BCS then assigned to groups of 10 (*n* = 9) for CONTROL, SPINT, and SPCROP winter pasture systems. Once assigned to a treatment, cows remained in the treatment unless they failed to breed or failed to wean a calf (calf death or injury) in which case they were removed from the study and replaced with a cow of similar age, breed type, BW, and BCS. In some instances, a cow that failed to wean a calf, but was reproductively sound, was bred and could return to the study as a replacement the following year. Table 5 summarizes the cows replaced by treatment and the reason for replacement. Following the approach used by Beck et al. (2016), treatment groups were co-mingled between replicates for the breeding season (May to July; due to this choice, the experiment was not designed to measure the effect of treatments on reproductive success). From the end of the breeding period in July to the start of breeding in May each year, cows remained in their assigned treatment replications. Cows were stocked on the paddocks at a rate of 0.62 cow-calf pairs ha^−1^ (10 cow-calf pairs per paddock per year, 30 cows per system per year).

**Table 5.** Percentages of cows replaced each year

Reason for replacement	Control	SPCROP	SPINT
Death or infirmity of cow	0.00%	0.00%	2.50%
Death or infirmity of calf	3.33%	0.83%	0.00%
Open cow	7.50%	5.00%	10.00%

Veterinary practices (Table 6) were completed following beef quality assurance protocols. In all years, pregnancy was determined via ultrasound. If pregnancy status was unable to be determined via ultrasound, blood samples were drawn and analyzed for the presence of pregnancy-specific glycoproteins.

**Table 6.** Cowherd health protocol during pre-breeding and weaning

Pre-breeding				
Year	Internal parasites	External parasites	Respiratory and reproductive	Clostridial
2016	LongRange^*^ (eprinomectin)	Ultra Saber^†^ (lambda-cyhalothrin)	CattleMaster 4 + VL5^‡^	
2017	LongRange^*^ (eprinomectin)	Ultra Saber^†^ (lambda-cyhalothrin)	Bovishield Gold FP5 + VL5^‡^	Calvary 9^2^
2018	LongRange^*^ (eprinomectin)	Ultra Saber^†^ (lambda-cyhalothrin)	Bovishield Gold FP5 + VL5^‡^	Calvary 9^†^
2019	LongRange^*^	StandGuard^∥^ (gamma-cyhalothrin)	CattleMaster 4 + VL5^‡^	Calvary 9^†^
Weaning				
Year	Internal Parasites	External Parasites	Respiratory and Reproductive	Clostridial
2016	Valbazen^‡^ (benzimidazole)	Cylence^$^ (cyfluthrin)		
2017			CattleMaster 4 + VL5^‡^	Bovilis 20/20 Vision 7 with Spur^†^
2018			CattleMaster 4 + VL5^‡^	
2019		Ultra Saber^†^ (lambda-cyhalothrin, piperonyl butoxide)	CattleMaster 4 + VL5^‡^	

^*^Merial Animal Health, Duluth, Georgia, USA.

^†^Merck Animal Health, Kenilworth, New Jersey, USA.

^‡^Zoetis, Parsippany-Troy Hills, New Jersey, USA.

^∥^Elanco, Greenfield, Indiana, USA.

^$^Bayer Animal Health, Shawnee, Kansas, USA.

veterinary practices were completed following Beef Quality Assurance (BQA) protocols and under veterinary guidance. All products were administered according to label directions.

Breeding bull veterinary practices followed the same chronology and protocol as cow health practices, with the exception of no reproductive synchronization. Bulls underwent and had to pass a breeding soundness examination yearly prior to turnout.

Each year at calving, calves were weighed, vaccinated, and bulls knife castrated. Calf veterinary practices followed beef quality assurance protocols. At birth calves were given a vaccine for infectious bovine rhinotracheitis-virus (IBR), and diarrhea parainfluenza-respiratory syncytial virus (BRSV; Bovi Shield Gold 5, Zoetis Inc., Kalamazoo, MI, USA).

### Forage Management

Forage mass (DM ha^−1^) was measured prior to cattle being moved to a new grazing area. Cattle were moved when approximately 60% of the total forage mass of the pasture had been removed. Forage mass was monitored using a monthly calibrated rising plate meter (Jennquip EC09—Jennquip—Feilding, New Zealand) using regression calibration equations developed for each system. Calibration was done by taking 30 clipped measurements from a 38.1 cm by 38.1 cm quadrant frame. The regression calibration equations were developed from these clipped measurements using procedures described by Cho, et al. (2019).

In all systems, hay and cube supplementation began when bermudagrass DM declined below a forage mass of 1,121 kg ha^−1^ (below approximately 15.25 cm canopy height). Forage nutritive value of hay (i.e., crude protein [CP], TDN, neutral detergent fiber, ADF) and stage of gestation determined the amount of supplementation required (NRC, 2016). Hand grab samples of forage within paddocks were sampled through the feeding period and analyzed for nutritive value. Samples were air dried at 53 °C to a constant weight for dry matter determination then samples were ground in a Wiley mill (Thomas Model 4 Wiley Laboratory Mill, Thomas Scientific, Swedesboro, NJ) to pass through a 1-mm screen. Samples were then scanned using the Foss 6500 spectrometer (Foss NIRSystems, Laurel, MD). Prediction equations developed from the Feed Testing Consortium (Hillsboro, WI) were used to predict CP, acid detergent fiber (ADF), and neutral detergent fiber. The total digestible nutrient (TDN) values were calculated with the Penn State equation: TDN = 4.898 + (89.796 × NEL) where NEL (Mcal/lb) is calculated as 1.0876 − (0.0127 ADF). Validation statistics for the equations used are provided in Table 7.

**Table 7.** Validation statistics for the Feed Testing Consortium equation used to predict the nutritive value of paddock grab samples

Constituent	*N*	SD^*^	*r* ^*2*^	SECV^†^
DM	106	1.50	0.975	0.311
CP	1,100	6.50	0.977	1.044
ADF	738	7.50	0.944	1.920
NDF	957	12.09	0.969	2.300

^*^Standard deviation.

^†^Standard error of validation.

**Figure 1.**
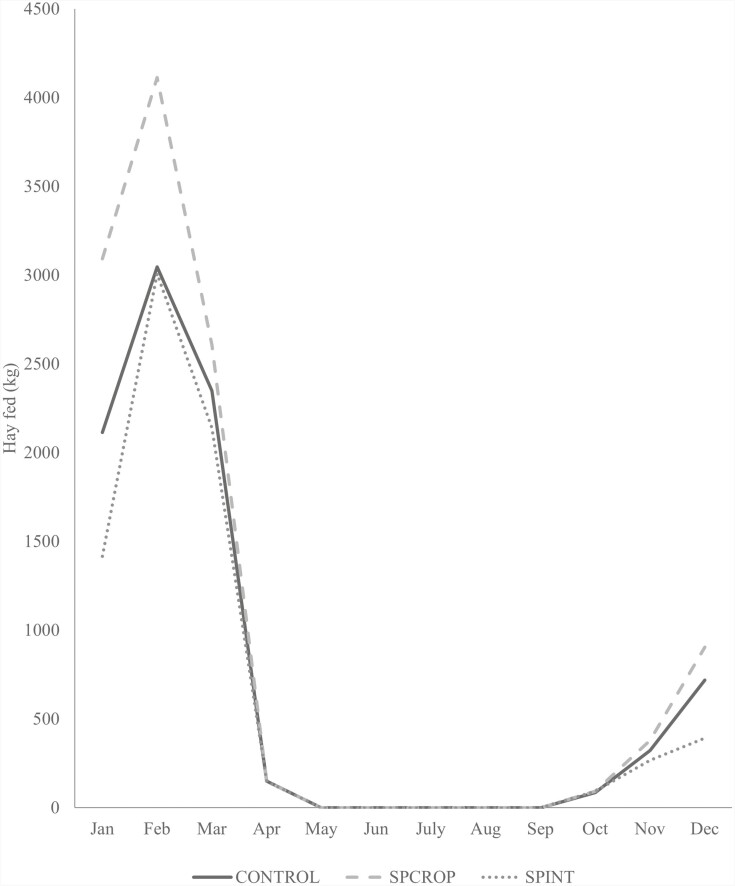
Average quantity of hay fed by month and grazing system. CONTROL is bermudagrass pasture and conventional hay and cube feeding; SPINT bermudagrass pasture with bermudagrass stockpile and interseeded wheat; SPCROP is bermudagrass with bermudagrass stockpile and cropland summer and winter pasture.

**Figure 2.**
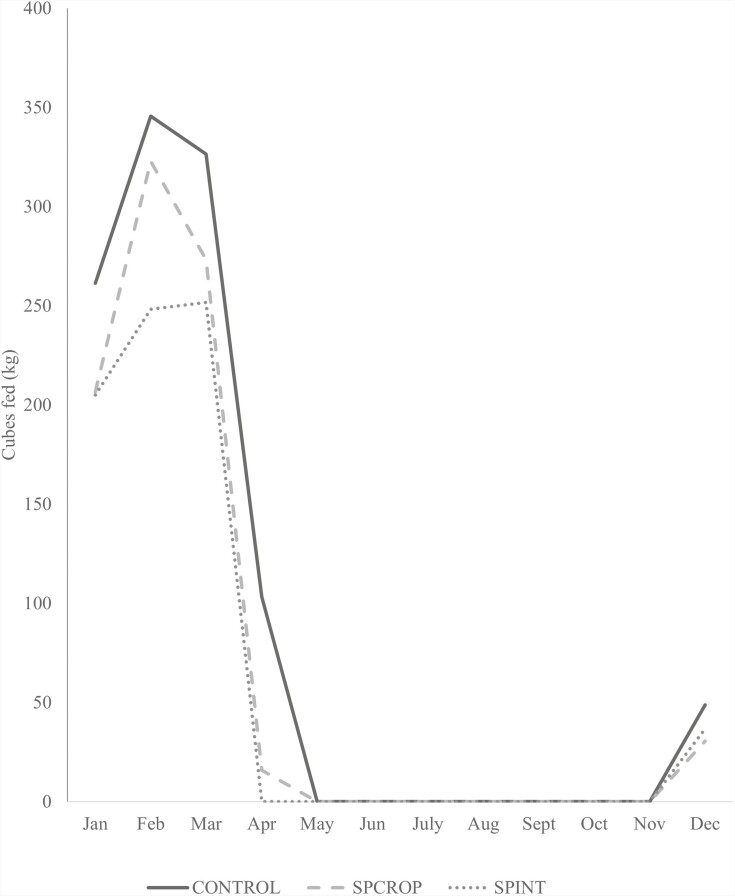
Average quantity of protein cubes fed by month and grazing system. CONTROL is bermudagrass pasture and conventional hay and cube feeding; SPINT bermudagrass pasture with bermudagrass stockpile and interseeded wheat; SPCROP is bermudagrass with bermudagrass stockpile and cropland summer and winter pasture.

Fed hay was purchased from trusted sources located near the study site and weights of fed bales were recorded prior to feeding. Hay and cube supplementation continued until bermudagrass forage mass was greater than 1,121 kg ha^−1^ DM (above approximately 15.25 cm canopy height). Hay feeding began in October, peaked in February, and then steadily declined until bermudagrass forage mass was sufficient to support grazing in May (Figure 1). Cube feeding was similar, with feeding beginning in November, increasing steadily to a peak in February, and then declining until cube feeding ceased in May which corresponded with fresh forage growth of sufficient nutrient content to support animal requirements (Figure 2).

Hectares devoted to bermudagrass stockpile were grazed short (approximately 10 cm height) by mid-August of each year. Stockpile areas were then fertilized with N and deferred from grazing to allow fresh stockpile to accumulate until the first killing frost (mid-November). Stockpile bermudagrass paddocks were grazed until a utilization level of 65% had been obtained (15 cm height) by monitoring with a rising plate meter and bermudagrass leaves had been removed.

Interseeded pasture was grazed until the emergence of winter wheat. At emergence, grazing was deferred to allow for winter pasture establishment. Interseeded pasture grazing began when the total DM ha^−1^ forage mass accumulated at least 1,345 kg ha^−1^. Once this forage mass threshold was reached, the paddocks were grazed until the wheat forage mass was depleted in April. Prior to the continuous stocking wheat pasture, cows were ration-stocked to wheat for approximately four hours per day as a supplemental protein source. After four hours cows were removed and fed hay.

Residual CC in the SPCROP system was terminated prior to establishing wheat in mid-September. Cropland wheat was allowed to emerge and establish a forage mass DM ha^−1^ threshold of at least 1,345 kg ha^−1^ before grazing was initiated. After which, cropland was grazed for 4 h every third day. On days cattle were not grazing wheat pasture, they returned to bermudagrass pasture and were supplied hay and cube supplements. At the end of the winter grazing season (April to May), cattle were allowed to completely graze wheat before CC establishment. CC were no-till seeded into cropland hectares and grazing began when forage mass reached 1,345 kg ha^−1^. Cows were allowed to graze the CC to a 50% utilization rate. This utilization rate was chosen to return some residue to maintain soil health.

### Statistical Analysis

Statistical analyses of the collected data were conducted using the MIXED Procedure in SAS (version 9.4; SAS Institute, 2012). One-way analysis of variance (ANOVA) was used to estimate the effect of treatments on measures of animal performance (calf BWT, calf weaning weight, and cow BCS prior to breeding) and feed (kg of hay and kg of cubes fed per month) variables. Differences of least squares means were compared using least significant difference tests and separated using the pdmix800 integrated macro program (Saxton, 1998). The model is represented as


$$\begin{array}{l} y_{st}=\mu_{s}+\tau_{t}+\varepsilon_{st} \end{array}$$ (1)

where $y_{st}$ represents the response variable in system s and year t for each animal performance variable (i.e., BWT, calf weaning weight, and cow BCS prior to breeding); $\mu_{s}$ is a system fixed effect where s∈ {CONTROL, SPINT, SPCROP}; $\tau_{t}$ is a year random effect where $\tau_{t}∼N(0,\sigma^{2})$; and $\varepsilon_{st}$ is the error term where $\varepsilon_{st}∼N(0,\sigma^{2})$. Likelihood ratio tests revealed there was a random year effect associated with the animal performance variables at the 95% confidence level. To account for the potential of period 1 grazing effecting forage mass in period 2 (i.e., serial correlation) on the experimental units (pastures), a repeated measure following an AR (1) process was included in the mixed modeling analysis. A likelihood ratio test verified the presence of serial correlation at a 95% level of confidence.

Feeding variables include the total kg of hay and kg of cubes fed per month in each system. The ANOVA is similar to Equation 1 except monthly data were used:


$$\begin{array}{l} f_{smt}=\gamma_{s}+\theta_{mt}+\varepsilon_{smt} \end{array}$$ (2)

where $f_{smt}$ represents the response variable of system s, month m, and year t for the feeding variables (total kg hay and cubes fed); $\gamma_{s}$ is a system fixed effect; $\theta_{mt}$ is a month × year random effect where $\theta_{mt}∼N(0,\sigma^{2})$; and $\varepsilon_{smt}∼N(0,\sigma^{2})$.

### Economic Methods

Enterprise budgeting was used to calculate expected revenues, costs, and net return for each grazing system (AAEA, 2000). Revenues were calculated as the least squares mean weaning weight multiplied by the price of produced heifers and steers in $ kg^−1^ (BIF, 2018). Calf prices for the 4-yr average wean date of October 8 (range October 3 to 13) were obtained from 10 yr (2011 to 2020) of Oklahoma City National Stockyards sales data for medium to large number one steers and heifers (USDA-AMS, 2020). Calves at weaning had a 4-yr average age of 219 d, ranging between 166 and 246 d. All sales prices were linearly interpolated using a price slide for a better representation of received prices in $ kg^−1^ with increasing weights. The price slide associated with steers and heifers was $−0.002 kg^−1^. All calves were assumed sold at weaning.

Input prices were obtained from local input supply dealers in January 2022. Paddocks were assumed to be in close proximity so, feeding cubes was assumed to be 6 min, while feeding one large round bale required 10 min of labor. Custom rates for fertilization, spraying, and no-till were from 2020 Oklahoma State University statewide averages (Sahs, 2020). To find proportionate fuel, lube, repair, labor, and fixed machinery costs, custom rates were decomposed based on known percentages in no-till establishment practices. Of the total custom rates 14% were allocated to labor, 46% to fuel, lube, and repairs, and 40% to fixed machinery depreciation expenses. Chemical product prices were based on the brand name product used in the experiment. Fertilizer prices were based on the applied sources of N, P, K, and lime. Fertilizer and soil amendment quantities applied were based upon real application quantities of N (as a mobile nutrient dependent upon the system) and an average application ha^−1^ of P and K. Lime applications were based upon a known ratio of 13/4 lime to N to keep soil pH constant (Havilin et al., 2005). To account for the current high fertilization costs, fertilizer (N, P, and K) prices were sourced from USDA ERS average U.S. farm prices of selected fertilizers. Twenty-year average (1995 to 2014) U.S. fertilizer prices revealed that current N, P, and K prices have increased by 150%, 217%, and 132%, respectively, (USDA, 2019^a^). The 20-yr average fertilizer prices were used in the economic analysis. Operating costs were subjected to a 5.5 percent interest rate to calculate the opportunity costs of capital for each system.

Economic results of tested treatments are likely sensitive to *ceteris paribus* changes in input prices and labor requirements. As a result, sensitivity analysis was conducted on variables that differ by system. Variables of interest included the cost of N, the cost of feed (hay and cubes), the wage rate, and feeding labor requirements for hay and cubes. N prices were changed by + 30%, −30%, and + 70% of base-case prices. The high price of N relative to previous years, an increase of 70% represents a scenario similar to prices in early 2022. The 10-yr average price of dry hay (2011 to 2020) did not reveal any change in the price of hay from the current price (USDA, 2019b). To account for price uncertainty in hay and cube production inputs, potential hay and cube price changes of + 30%, −30%, and + 50% from base-case prices were considered. Wage rates were changed by + 30%, −30%, and −50% from base-case hourly wages to represent additional or reduced feeding and machinery labor costs. Labor assumptions for feeding protein cubes were increased from 6 min to 10, 15, and 20 min per feeding. Labor requirements for feeding hay were increased from 10 min to 15, 20, and 25 min per 1.52 × 1.83 m round bale. This analysis represents the possibility of different farms having additional travel distances to storage facilities and between paddocks.

## Results and Discussion

### Forage Mass and Quality

Forage mass (kg ha^−1^) by month and for each production system is reported in Table 8. Forage mass was similar between systems during the fall months of October and November. In response to colder weather, bermudagrass forage growth for all three systems declined. However, the wheat growth in the SPINT paddocks during the winter months, increased the relative average biomass for that system compared to the other two, achieving the desired agronomic effect of the SPINT systems. As warmer weather increased in March and April, the average forage mass of interseeded cool-season annuals declined. By April, bermudagrass forage growth between all three systems was similar.

**Table 8.** Monthly mean forage mass by grazing system, kg ha^−1^

	Grazing system^*^		
Month	CONTROL	SPCROP	SPINT
Oct.	2,175	2,125	2,359
Nov.	1,970	2,146	2,410
Dec.	1,865^B^	1,640^b^	2,285^aAb^
Jan.	1,730^b^	1,640^b^	2,239^a^
Feb.	1,653^b^	1,426^b^	2,249^a^
Mar.	1,586^b^	1,401^b^	2,063^a^
Apr.	1,820	1,704	2,066

^*^CONTROL is bermudagrass pasture and conventional hay and cube feeding; SPINT bermudagrass pasture with bermudagrass stockpile and interseeded wheat; SPCROP is bermudagrass with bermudagrass stockpile and cropland summer and winter pasture

^a,b,A,B^Lowercase letters that differ within a row (month) are significantly different at a 95% level of confidence, and uppercase letters that differ within a row are significantly different at a 90% level of confidence.

## Animal Performance

Overall, calf BWT (Table 9) was not significantly different (*P *= 0.2733), with a range of 1.21 kg. Weaning weight between systems was also not statistically different (*P* = 0.6936). The increased calf weights associated with creep grazing exhibited by Apple et al. (1991) and Apple et al. (1993) were associated with fall-calving cows. In the experiment reported here, spring calves did not have access to the cool-season forage due to being sold before the availability of wheat pasture. Calf BW increases would be more likely in fall-calving cows. This also differs slightly from the study by Beck et al. (2016), which focused on the stocking rate in bermudagrass stockpiling systems with complementary cool-season forages in spring-calving operations. Beck et al. concluded that the more intensive stockpiling and cool-season forages produced calf weaning weights that were significantly lower than continuous grazing without additional grazing extension practices. Cows at higher stocking rates on stockpiled forage typically had lower BCS, and as such this was reflected in calf weaning weights. The difference in the studies could be that cows were fed here following the goal of maintaining the same body condition across treatments.

**Table 9.** Least square means and standard errors for cow-calf production and feed variables

	Grazing system^*^						
	CONTROL		SPINT		SPCROP		
Variable of interest	Mean	S.E.M.	Mean	S.E.M.	Mean	S.E.M.	*P*-value
*Calves* ^†^							
Calf birth weight, kg	40.35	0.83	39.6	0.84	39.14	0.84	0.2733
Weight prior to breeding dam, kg	111.89	3.03	111.28	3	109.83	3.01	0.735
Weaning weight, kg	273.37	2.98	274.69	2.94	276.91	2.94	0.6936
*Cows*							
Body condition score before breeding	6.81	0.12	6.97	0.12	7.04	0.12	0.0727
*Amount of feed required*							
Cubes fed per month, kg hd^−1^	21.71A	3.38	14.84B	3.38	16.98B	3.38	0.0001
Hay fed per month, kg hd^−1^	125.50B	29.71	106.67B	29.71	161.98A	29.71	0.0048

^*^CONTROL is bermudagrass pasture and conventional hay and cube feeding; SPINT bermudagrass pasture with bermudagrass stockpile and interseeded wheat; SPCROP is bermudagrass with bermudagrass stockpile and cropland summer and winter pasture.

^†^Least squares means of calf and cow variables were based upon 10 hd per replication.

Cows had similar BCS prior to breeding (*P* = 0.0727) at the 95% confidence level, but were different at a 90% confidence level. In each system, BCS was above ideal levels (5 to 6), making them over-conditioned at breeding (Walker, 2017). This would suggest that as the study progressed, stocking rate could have increased in all treatments. Although high, similar BCS affirms that the combination of forage and supplementary hay and cubes would have similar potential re-breeding success.

Additional feed through hay and range cube supplementation varied between systems. Total kg of cubes fed to cows in each pasture (replication) per month were highest in the CONTROL system (*P *< 0.0001) at 21.71 kg and numerically higher in the SPCROP system relative to the SPINT system, with 16.98 and 14.84 kg fed, respectively. Because the SPCROP and SPINT systems allowed cattle to graze winter wheat pasture, which has a relatively high concentration of CP, the need for increased protein-based supplement was lower. This is consistent with the study by Apple et al. (1991), Apple et al. (1993), Dillard et al. (2018), Hoveland et al. (1978), Rouquette (2017), and Mullenix and Rouquette (2017).

Total hay fed to cows in each pasture (replication) per month was highest in the SPCROP system (*P* = 0.0048) at 161.98 kg. A total of 125.50 kg of hay was fed per month in the CONTROL system, which was numerically higher than cattle fed in the SPINT system which used 106.67 kg of fed hay per month. Even though wheat pasture has high CP, the reduction of 3 ha of pastureland in the SPCROP system reduced potential forage through dormant bermudagrass. As such, additional protein from cubes was not needed, so cattle were fed hay to meet energy requirements.

## Economics

With little variation in animal performance, economic results were determined by differences in costs. Sources of average per hectare revenues, costs, and associated net returns are in Table 10. Sources of revenue were limited to the sale of calves produced by each system. Because the least squares mean of calf weaning weight did not differ by treatment, the total kg ha^−1^ also did not differ. Steers and heifers received prices of $3.73 kg^−1^ and $3.51 kg^−1^, respectively. The average price received for calves, regardless of sex, was $3.62 kg^−1^. Due to such small differences in weights, the price slide was not used. Gross revenue was $613 ha^−1^, $616 ha^−1^, and $621 ha^−1^ for the CONTROL, SPCROP, and SPINT systems, respectively. Overall, the factors that decided the most economical system were the costs associated with seed, feed, machinery, and fertilizer costs.

**Table 10:** Calving production, sources of revenues, production costs, and net returns to land, management, and farm overhead by grazing system

	Grazing system^*^		
Animal performance and economic variables	CONTROL	SPINT	SPCROP
*Sources of revenue*			
Average calf weight, kg ha^−1^	154.48	151.91	154.02
Average price received, $ kg^−1^	3.62	3.62	3.62
Gross revenue from calves, $ ha^−1^	612.81	615.75	620.74
*Production costs*			
Herbicide prior to planting/interseeding, $ ha^−1^	—	1.46	2.20
Herbicide to control broadleaf weeds, $ ha^−1^	22.41	22.44	18.58
Cow, calf, and bull health/veterinary practices, $ ha^−1^	26.19	26.17	26.42
Small grain seed, $ ha^−1^	—	18.53	12.97
Cover crop seed, $ ha^−1^	—	—	12.97
Hay and cubes, $ ha^−1^	151.77	118.22	167.81
Hay and cubes: labor for feeding, $ ha^−1^	18.48	14.21	18.98
Cost of fencing for rotational stocking, $ ha^−1^	34.87	34.87	52.29
Cost of water for rotational stocking, $ ha^−1^	21.25	21.25	21.25
Machinery labor, $ ha^−1^	4.37	6.47	7.86
Machinery fuel, $ ha^−1^	14.36	21.28	25.80
Fertilizer (N, P, K, and. Lime), $ ha^−1^	70.10	79.00	83.89
Total cash operating expenses, $ ha^−1^	363.84	363.89	455.66
Interest on operating capita, $ ha^−1^	20.01	20.01	25.06
Breeding bulls fixed ownership costs, $ ha^−1^	49.42	49.42	49.42
Machinery fixed ownership cost, $ ha^−1^	12.48	18.51	22.44
Total cost, $ ha^−1^	445.75	451.83	552.58
Total cost, $ hd^−1^	275.37	279.13	341.37
Net returns to land, management, and overhead, $ ha^−1^	167.06	163.92	68.15
Net returns to land, management, and overhead, $ hd^−1^	103.21	101.21	42.10
Difference in net returns against best system, $ ha^−1^	0.00	−3.13	−98.90
Difference in net returns against best system, $ hd^−1^	0.00	−1.94	−61.10

^*^CONTROL is bermudagrass pasture and conventional hay and cube feeding; SPINT bermudagrass pasture with bermudagrass stockpile and interseeded wheat; SPCROP is bermudagrass with bermudagrass stockpile and cropland summer and winter pasture.

The returns in the table are per calf weaned and are not adjusted for reproductive failures. While reproductive failure is important economically (Boyer et al. 2020), the experiment was not designed to measure the effects of the treatments on reproductive failure.

The CONTROL system did not require winter small grain or summer CC seeding, so it had no seed costs. Both SPINT and SPCROP systems used winter wheat seed for wheat pasture and annual ryegrass seed for the stockpile, but the SPCROP system required 1 ha less winter seed, resulting in the SPINT system being $6 ha^−1^ more costly for cool-season seeding. The CC seed for the SPCROP system cost $13 ha^−1^. The SPCROP system had an average cost of $26 ha^−1^ for winter and summer seeds. This is $7 ha^−1^ more than the SPINT system and $26 ha^−1^ higher than the CONTROL system.

Results of the bermudagrass stockpile, cropland, and winter wheat interseeded systems on reducing feed costs were mixed. Feeding costs were highest in the SPCROP system at $168 ha^−1^. The SPINT system had the lowest feed cost of $118 ha^−1^, while the CONTROL system had feed costs slightly less than the SPCROP system at $152 ha^−1^. Therefore, the feeding costs in SPCROP were $16 and $50 ha^−1^ higher than the CONTROL and SPINT systems, respectively. The cost of feeding labor mirrored the feed costs with the SPCROP, CONTROL, and SPINT systems requiring $19, $18, and $14 ha^−1^ in labor, respectively.

Because the SPCROP and SPINT systems required more seed, fertilizer and pesticides, they required more machinery usage, and thus more machinery costs. The SPCROP system had the most machinery usage. The SPCROP system had the most machinery usage. Relative to SPINT system and the CONTROL, respectively, the SPCROP system resulted in a $2 and $4 ha^−1^ increase in labor charges, a $5 and 12 ha^−1^ increase in fuel charges, and a $3 and $10 ha^−1^ increase in fixed machinery ownership costs. When summed together, the SPCROP system’s machinery costs were $56 ha^−1^, the SPINT system’s costs were $46 ha^−1^, and the CONTROL system’s machinery costs were $31 ha^−1^.

Fertilizer costs accounted for 19%, 22%, and 18% of all cash costs in the CONTROL, SPINT, and SPCROP systems, respectively. Differences in cost were most evident in the N and lime applications. The SPCROP system required the most fertilization and soil amendment applications at a cost of $84 ha^−1^. This was $5 ha^−1^ higher than the SPINT system and $14 ha^−1^ higher than the CONTROL system.

Total costs, including average annual fixed machinery ownership costs and breeding stock ownership costs, were $446 ha^−1^ ($275 hd^−1^), $452 ha^−1^ ($279 hd^−1^), and $553 ha^−1^ (341 hd^−1^) for the CONTROL, SPINT, and SPCROP systems.

Net returns to land, management, and overhead were $167 ha^−1^ ($103 hd^−1^), $164 ha^−1^ ($101 hd^−1^), and $68 ha^−1^ ($42 hd^−1^) for the CONTROL, SPINT, and SPCROP systems. Costs incurred were highest in the SPCROP system, but relatively similar in the CONTROL and SPINT systems, with the SPINT system being $6 ha^−1^ ($3.76 hd^−1^) more costly. Overall, the CONTROL system was the most economical, realizing a net return of $3 ha^−1^ ($1.94 hd^−1^) higher than the SPINT system and $99 ha^−1^ ($61 hd^−1^) higher than the SPCROP system.

Changes in input prices could result in changes in the economically preferred system. The results of the sensitivity analysis presented in Table 11 are consistent across a variety of price and feeding labor time scenarios. The difference in net returns between the CONTROL and SPINT systems is small and so their ranking is sensitive to the changes in assumptions. With the much larger gaps in net returns for the SPCROP system, there is no scenario where the SPCROP system was preferred. While a SPCROP system might work for a stocker cattle operation (Key et al. 2023), it was too costly relative to returns for the cow-calf grazing systems considered here.

**Table 11.** Expected net returns, $ ha^−1^, for *ceteris paribus* changes in per unit prices of fertilizer, hay, protein cubes, labor, and time requirements for feeding hay and protein cubes

			Grazing system^*^		
Production input	Sensitivity scenario	Unit	CONTROL	SPINT	SPCROP
Price of N (46-0-0), $ kg^−1^	Base-case	0.88	167.06	163.92	68.15
	Base-case −30%	0.62	184.63	180.36	88.38
	Base-case + 30%	1.15	149.47	147.51	47.91
	Base-case + 70%	1.50	126.03	125.62	20.93
Price of hay, $ kg^−1^	Base-case	0.18	167.06	163.92	68.15
	Base-case −30%	0.12	196.64	189.40	107.69
	Base-case + 30%	0.23	137.46	138.47	28.59
	Base-case + 50%	0.26	117.73	121.49	2.23
Price of cubes, $ kg^−1^	Base-case	0.87	167.06	163.92	68.15
	Base-case −30%	0.61	176.50	175.88	81.71
	Base-case + 30%	1.13	148.60	151.99	54.58
	Base-case + 50%	1.30	136.30	144.03	45.54
Price of labor, $ hr^−^^1^	Base-case	15.00	167.06	163.92	68.15
	Base-case + 30%	19.50	159.81	157.39	59.66
	Base-case −30%	10.50	174.28	170.48	76.63
	Base-case −50%	7.50	179.11	174.85	82.29
Labor feeding hay, min	Base-case	10	167.06	163.92	68.15
	Base-case + 50%	15	161.86	159.66	61.49
	Base-case + 100%	20	156.66	155.38	54.83
	Base-case + 150%	25	151.47	151.10	48.17
Labor feeding cubes, min	Base-case	6	167.06	163.92	68.15
	Base-case + 67%	10	160.97	159.65	63.68
	Base-case + 150%	15	153.36	154.30	58.10
	Base-case + 233%	20	145.76	148.94	52.52

^*^CONTROL is bermudagrass pasture and conventional hay and cube feeding; SPINT bermudagrass pasture with bermudagrass stockpile and interseeded wheat; SPCROP is bermudagrass with bermudagrass stockpile and cropland summer and winter pasture.

The budgets are based on assuming no differences in reproductive success across treatments. The experiment was designed so that every cow weaned a calf and so the returns in the table are not adjusted for reproductive failures.

## Conclusions

Animal performance results suggested a lower need for protein cubes for a system that allows grazing wheat during the cool-season months when bermudagrass pastures were dormant. Moreover, the additional costs of establishing seeded pastures, both in cropland and interseeded into bermudagrass pasture, did not produce enough additional revenue or feed cost savings to support their use compared to the conventional system. The more intensive winter/summer annual and stockpiling systems required higher fertilization and machinery use, resulting in a higher total cost. Finally, the increased dry matter availability through stockpile grazing was not enough to reduce feed costs to make it more attractive economically compared to the common system. Overall, the relative results were not overly sensitive to incremental changes in the prices of inputs or labor requirements for feeding hay and cubes.

Due to the limited number of experimental units (pastures) available for this study, adding an additional treatment for different stocking rates was not possible. Future studies that include stocking rates can add value to this study to help understand the economics of the two alternative systems during periods of relatively wet summers where excess forage biomass is available. In addition, only enough pastures were available to evaluate two alternative production systems. Evaluation of different forage species to interseed into perennial bermudagrass pastures and different CC mixes established for summer grazing on the cropland acres might improve the economics for those systems, respectively. Furthermore, this study only used one level of feed-to-bodyweight ratio. Evaluation of alternative feed-to-bodyweight ratios might improve the economics of feeding practices in the winter months.
